# Synthesis and characterization of Zn and Fe doped magnetic biochar from *Acacia falcata* leaves for Cr(VI) adsorption

**DOI:** 10.1038/s41598-025-06319-9

**Published:** 2025-07-01

**Authors:** Rajesh Juturu, Ramesh Vinayagam, Gokulakrishnan Murugesan, Raja Selvaraj

**Affiliations:** 1 Department of Biotechnology, Manipal Institute of Technology, Manipal Academy of Higher Education, Manipal, Karnataka 576104 India; 2 Department of Chemical Engineering, Manipal Institute of Technology, Manipal Academy of Higher Education, Manipal, 576104 Karnataka India; 3 Department of Biotechnology, M.S. Ramaiah Institute of Technology, Bengaluru, Karnataka 560054 India

**Keywords:** Adsorption, Superparamagnetic, Cr(VI) removal, Magnetic Biochar, Electrostatic attraction & reduction, Environmental sciences, Mathematics and computing, Nanoscale materials, Chemical engineering, Chemical engineering, Environmental chemistry, Green chemistry, Materials chemistry

## Abstract

Hexavalent chromium (Cr(VI)), a toxic pollutant extensively utilized across multiple industries, necessitates effective treatment using low-cost and sustainable materials. In this research, magnetic biochar (MBC) was prepared using *Acacia falcata* leaves through chemical treatment with ZnCl_2_ and incorporating Fe_3_O_4_ nanoparticles using FeCl_3_·6H_2_O as a precursor. BET analysis revealed a specific surface area of 248.11 m^2^/g and FESEM images showed a highly porous structure with uniformly embedded Fe_3_O_4_ nanoparticles, which became smoother and more compact after Cr(VI) removal. XRD spectra confirmed the incorporation of Fe_3_O_4_ through distinct (311) and (440) peaks, matching the cubic spinel structure, while VSM data revealed a magnetic saturation of 5.44 emu/g. XPS analysis indicated the participation of carboxyl, hydroxyl, carbonyl, and Fe_3_O_4_ groups in Cr(VI) reduction and adsorption. Batch experiments identified an optimum pH of 2, a MBC dose of 0.4 g/L, and a contact time of 3 h. The adsorption data followed the Freundlich isotherm and pseudo-second-order kinetics. The maximum adsorption capacity was 25.62 mg/g at 303 K, and thermodynamic studies confirmed that Cr(VI) removal was spontaneous and endothermic. The enthalpy and entropy values for Cr(VI) adsorption were 17.71 kJ/mol and 63.22 J/mol·K, respectively. Reusability studies, conducted at the optimum pH of 2 and a MBC dose of 1.4 g/L, demonstrated that MBC could be reused for up to five cycles. MBC effectively removed over 97.80% of Cr(VI) from various water sources, highlighting its potential for Cr(VI) remediation.

## Introduction

Water pollution is increasingly linked to the rise in the standard of living, as higher consumption of goods leads to more waste production and industrial discharge. Various industries, including textile, leather tanning, electroplating, and chemical manufacturing, contribute significantly to water pollution by discharging heavy metal effluents. Amid these pollutants, hexavalent chromium (Cr(VI)) is a poisonous chemical and is extensively employed in multiple industries. Cr(VI) dissolves readily and swiftly infiltrates biological membranes, posing severe risks to human beings, such as cancer, liver damage, and respiratory problems^1^. In aquatic environments, Cr(VI) is toxic to marine life, affecting reproduction and growth, and disrupting entire ecosystems. The persistence and bioaccumulation of chromium in the environment make it a critical pollutant requiring effective treatment methods.

Several techniques are available to treat Cr(VI) effluent, including ion exchange, membrane process, precipitation, electrochemical method, and adsorption^2^. Amongst them, adsorption is an easy, and effective process for eliminating trace Cr(VI) from aqueous media. This method works by capturing Cr(VI) species on the adsorbent’s surface, allowing regeneration and reuse while minimizing waste production. Moreover, adsorption avoids the generation of hazardous by-products, making it an environmentally sustainable option^3^. Utilizing low-cost, renewable adsorbents such as biomass-derived magnetic biochar further enhances its economic and environmental benefits, positioning adsorption as a highly effective solution for Cr(VI) remediation.

Various carbonaceous materials, consisting of activated carbon (AC), biochar, magnetic biochar (MBC), and nanoparticles, have been explored for Cr(VI) remediation. These materials are often modified with acids, bases, or polymers to enhance their surface properties, functional groups, and adsorption capacity. Among these, the magnetic biochar has gained attention due to its unique advantages. The incorporation of magnetic nanoparticles imparts magnetic properties, enabling effortless adsorbent recovery from water via a magnetic field^4^. Additionally, Fe^2+^/Fe^3+^ facilitates the reduction to Cr(III) state, improving Cr(VI) adsorption. Its large surface area, reusability, and ease of recovery make MBC an efficient and sustainable material for Cr(VI) remediation.

Chemical activation is a widely adopted method to enhance the properties of biochar for contaminant removal, as it significantly modifies the surface characteristics and functionality of the material. This process involves treating biomass with chemical agents, such as acids (H_3_PO_4_, H_2_SO_4_), bases (KOH, NaOH), or salts (ZnCl_2_, FeCl_3_), which promotes the development of a porous structure and introduces functional groups that are crucial for adsorption^5^. The incorporation of these activating agents increases the surface area and pore volume of biochar, thereby providing more active sites for contaminant binding. Among these, FeCl_3_ is a promising activating agent, as it imparts magnetic properties to biochar; however, it lacks effectiveness in pore formation and results in a lower surface area^6^. This limitation can be addressed by simultaneous activation with ZnCl_2_, which enhances surface area and porosity by breaking down organic matter during carbonization. Additionally, ZnCl_2_ promotes dehydration and aromatization, leading to a more ordered and structurally stable carbon material^7^. In recent years, Zn–Fe doped magnetic biochar has been successfully applied for the removal of various pollutants such as nitrobenzene^8^, lead^9^, chromium^10^, and tetracycline^11^. However, there is still a need to explore the synthesis of Zn–Fe doped magnetic biochar derived from diverse biomass sources for the effective removal of a broader range of contaminants.

In this study, *Acacia falcata*, commonly known as the sickle wattle, is a fast-growing tree recognized for its dense foliage and high lignocellulosic content, making it an ideal precursor for magnetic biochar synthesis. The leaves of *A. falcata* are rich in phytochemicals such as tannins, saponins, flavonoids, and polyphenols, which not only contribute to the carbon yield during pyrolysis but also introduce surface functionalities favorable for adsorption processes^12^. In the present study, *A. falcata* leaves were utilized as a sustainable and eco-friendly biomass source for the synthesis of Zn–Fe doped magnetic biochar. The novelty of this work lies in the simultaneous exploitation of naturally occurring phytochemicals in the leaves and the co-doping with metal ions to develop a cost-effective, magnetically responsive adsorbent for Cr(VI) remediation.

This study focuses on overcoming key drawbacks of existing adsorbents, such as limited adsorption capacity, high production costs, susceptibility to interference from coexisting ions, and poor reusability. The main objective of this study was to: (1) synthesize Zn–Fe doped magnetic biochar using *A. falcata* leaves as a sustainable precursor; (2) characterization of magnetic biochar using various techniques ; (3) evaluate the MBC’s performance under varying conditions, including pH, contact time, adsorbent dose and initial Cr(VI) concentration; (4) evaluation of the adsorption data using various kinetic, isotherm, and thermodynamic models; (5) elucidation of Cr(VI) removal mechanism; (6) regeneration assessment of MBC; (7) effect of coexisting ions present in water sources and their impact on the Cr(VI) removal.

## Experimental section

### Chemicals

*A. falcata* leaves were taken from trees located within the premises of the college campus. The collected leaves were rinsed to eliminate surface impurities and then dried to remove moisture before further processing. Sulfuric acid, ferric chloride hexahydrate, sodium hydroxide, zinc chloride, and hydrochloric acid, were procured from Loba Chemie, India. Additionally, acetone, potassium dichromate, and 1,5-diphenylcarbazide were obtained from Merck, India.

### Synthesis of magnetic Biochar

**Fig. 1:**
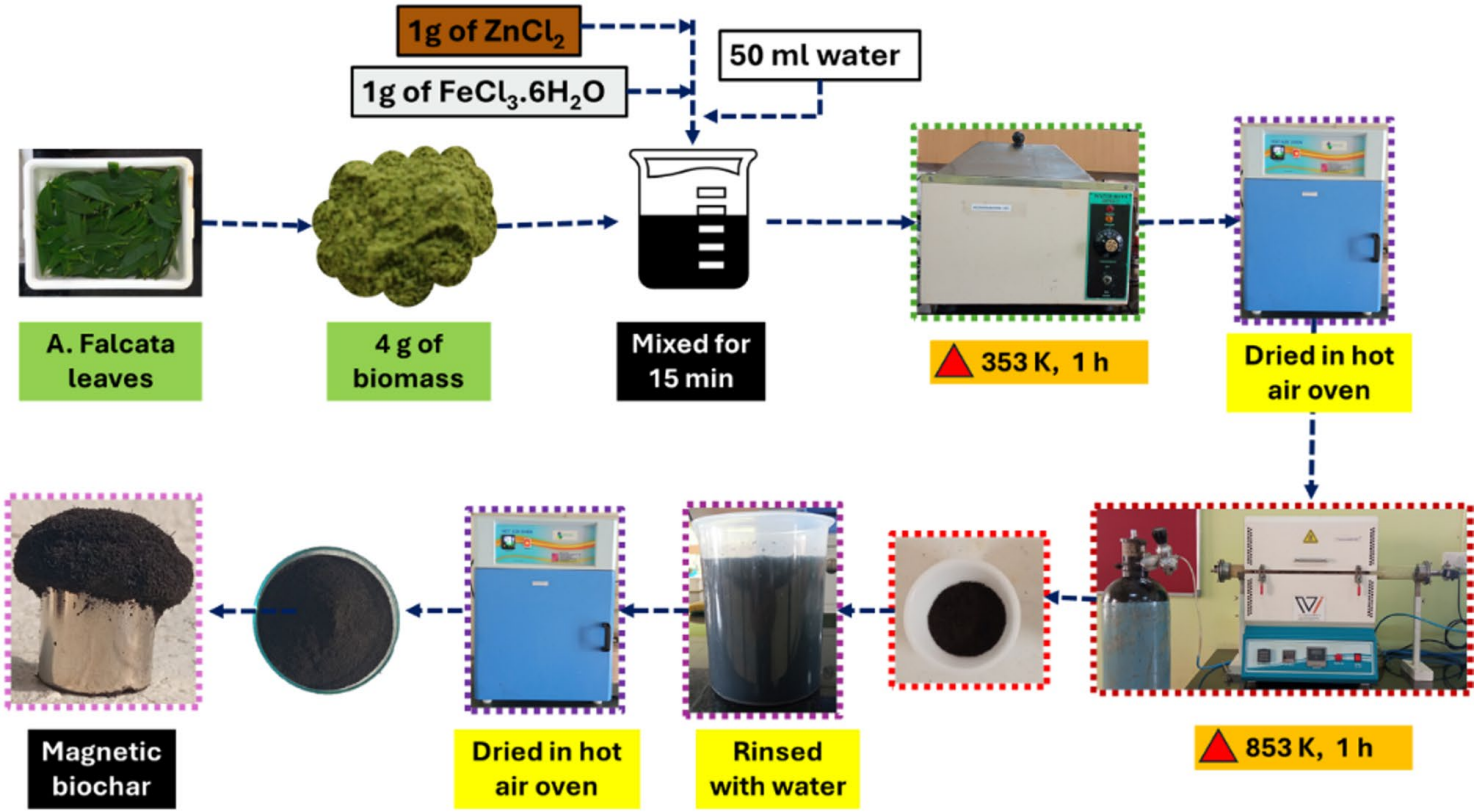
Schematic representation of the synthesis process of magnetic biochar derived from *A. falcata* leaves using a tubular furnace.

To prepare the magnetic biochar, 1 g of FeCl_3_.6H_2_O and 1 g of ZnCl_2_ were precisely measured and placed into a clean beaker. These components were mixed with distilled water (50 mL) and agitated employing a stirrer to ensure complete dissolution. Once a homogeneous solution was obtained, 4 g of *A.falcata* leaf powder was added to the beaker. The contents were agitated continuously to achieve uniform dispersion of the precursor solution onto the biomass surface. The prepared mixture was then subjected to heating at 353 K for 1 h using a water bath, allowing the precursors to impregnate the plant material effectively. Following this step, the material was oven-dried at 353 K. After drying, the contents were carefully transferred into ceramic crucibles and pyrolyzed at 873 K for 1 h in a nitrogen-purged tubular furnace. ZnCl_2_ primarily functions as a chemical activating agent, promoting porosity development in the carbon matrix through dehydration and the release of volatiles during pyrolysis. Simultaneously, FeCl_3_·6H_2_O undergoes hydrolysis followed by thermal decomposition, leading to the in-situ formation of iron oxides embedded within the carbon framework^5^. The high-temperature treatment not only enhances structural activation but also facilitates the formation of magnetic nanoparticles, ultimately yielding a magnetically responsive carbonaceous material. Post-carbonization, the obtained product was thoroughly rinsed with water to remove unreacted precursors. Following this step, the material was oven-dried to obtain the magnetic biochar (Fig. 1).

### Adsorbent characterization

The characterization of the synthesized MBC was conducted using various analytical methods to assess its morphological, elemental, and magnetic properties. The MBC’s composition and morphology were analyzed using EDS (Oxford Instrument, UK) and SEM (Neon 40, Carl Zeiss, Germany). The Brunauer-Emmett-Teller (BET) evaluation was carried out using Smart Instruments, India, to determine the pore volume, specific surface area, and porous nature. The material’s constituents and elemental states were analyzed using X-ray photoelectron spectroscopy (XPS) (K-Alpha 149, Thermo Fisher Scientific, UK). The functional moieties on the MBC surface were analyzed with Fourier Transform Infrared Spectroscopy (FTIR) with an instrument from Agilent Technologies, USA. D8 Advance instrument from Bruker, Germany, was used for X-ray diffraction (XRD) to examine the material’s phase structure and crystallinity. MBC’s magnetic behavior was analyzed using VSM 8600 (Lake Shore Cryotronics, US). Thermogravimetric analysis (TGA) was carried out using a PerkinElmer STA 6000. The sample was heated from room temperature to 800 °C at 10 °C/min under a nitrogen atmosphere.

### Cr(VI) adsorption experiments

The Cr(VI) removal efficiency of the synthesized MBC was evaluated through batch adsorption experiments. These studies were conducted in conical flasks, maintaining a constant temperature of 303 K, 100 ml of Cr(VI) solution, an adsorption time of 3 h, and an agitation speed of 150 rpm, unless otherwise specified. Cr(VI) concentrations before and after adsorption were measured using the standard 1,5-diphenylcarbazide (DPC) colorimetric method. Absorbance was recorded at 540 nm using a UV–Vis spectrophotometer (UV-Shimadzu 1900i), and Cr(VI) concentrations were determined. Each experiment was performed in triplicate to verify the result’s reliability and reproducibility. The removal efficiency (R) and adsorption capacity (q_e_) of the MBC were evaluated using Eqs. (1) and (2).

1 $$\:R\left(\%\right)=\frac{\left({\text{C}}_{\text{i}}-{\text{C}}_{\text{f}}\right)}{{\text{C}}_{\text{i}\text{n}\text{t}}}\times\:100$$

2 $$\:{q}_{e}=\frac{({\text{C}}_{\text{i}}-{\text{C}}_{\text{f}})}{{\text{m}}_{\text{M}\text{B}\text{C}}}\times\:\text{V}$$

where C_i_ and C_f_ denote the respective initial and final Cr(VI) concentrations, V is the volume of Cr(VI) feed, and $\:{\text{m}}_{\text{M}\text{B}\text{C}}$ is the mass of the MBC.

The influence of various operational conditions on Cr(VI) removal was examined by altering one parameter at a time while retaining the others constant. First, the effect of pH was examined by adjusting between 2 and 12 while maintaining a fixed MBC dose (0.4 g/L), 10 mg/L Cr(VI) concentration, and contact time (3 h). Next, the MBC dosage (0.1–0.9 g/L) was varied while retaining other parameters at the optimum pH. Subsequently, the Cr(VI) concentration (5–25 mg/L) and time (5–180 min) were changed while maintaining the optimum pH and dosage. Finally, the temperature (293–323 K) was varied, retaining other variables at optimal values. Experimental data were assessed by applying pseudo-first-order (PFO), pseudo-second-order (PSO), and intra-particle diffusion (IPD) models to establish the precise model for Cr(VI) remediation^13^. Isotherms were derived from Cr(VI) concentrations (5–25 mg/L) and analyzed using Freundlich, Langmuir, and Temkin models^14^. Additionally, adsorption experiments at 293, 303, 313, and 323 K were performed to analyze the thermodynamic spontaneity of the process.

### Regeneration studies

The reusability of magnetic biochar (MBC) was systematically evaluated through multiple adsorption-desorption cycles to assess its stability and performance over repeated use. In the initial cycle, batch adsorption experiments were carried out by treating 100 mL of a 10 mg/L Cr(VI) solution with 1.4 g/L of MBC to achieve 100% Cr(VI) removal. The solution pH was adjusted to 2 to maximize Cr(VI) uptake. The mixture was agitated at 150 rpm for 3 h in a shaker, and temperature maintained at 303 K.

Following the adsorption process, the Cr(VI)-loaded MBC was separated from the solution using an external magnet. The supernatant was carefully decanted and discarded, and the regeneration of the adsorbent was initiated by adding 50 mL of 0.1 N NaOH to the flask containing the spent MBC. The desorption step was conducted by agitating the mixture in an orbital shaker at 150 rpm for 2 h at 303 K to facilitate the release of adsorbed Cr(VI) from the MBC surface. After desorption, the MBC was washed thoroughly with deionized water, dried, and reused for the subsequent adsorption cycle under identical conditions.

### Cr(VI) adsorption from multiple water matrices

To evaluate the performance of magnetic biochar (MBC) for Cr(VI) removal in realistic environmental conditions, various water matrices including river water, lake water, groundwater, and tap water were collected. These matrices were each spiked with Cr(VI) to a final concentration of 10 mg/L using a prepared stock solution. Batch adsorption experiments were then conducted to assess the removal efficiency of MBC.

For each experiment, 100 mL of the Cr(VI)-spiked water was adjusted to an acidic pH of 2, which is known to favor the adsorption of hexavalent chromium. A MBC dosage of 1.4 g/L was added to the solution. The contents were agitated at 150 rpm using a rotary shaker at a constant temperature of 303 K. After 3 h of contact time, samples were withdrawn, and the residual Cr(VI) concentration was measured.

## Result and discussion

### MBC characterization results

**Fig. 2:**
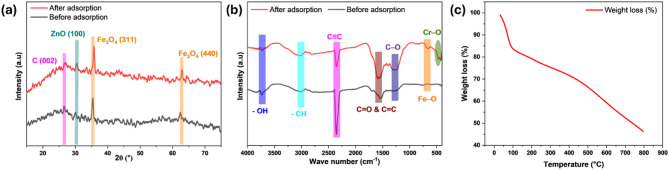
XRD (**a**) and FTIR (**b**) analyses of MBC before and after Cr(VI) adsorption, TGA (**c**) analysis of MBC.

Synthesized MBC XRD patterns are presented in Fig. 2a. The prominent 26.73° diffraction signal corresponds to the (002) plane of carbon, indicating the presence of graphitic carbon within the biochar matrix. This peak indicates disordered graphitic structures and amorphous carbon with short-range graphene layer stacking, confirming the successful carbonization of *A. falcata* leaves. The two distinct peaks at 2θ = 35.40° and 62.35°, associated with the (311) and (440) planes match the cubic spinel magnetite structure (JCPDS 19–0629), confirming the incorporation of Fe_3_O_4_ into the biochar^15^. The lattice parameter of 0.841 nm agrees with reported magnetite values, verifying its cubic spinel structure^16^. The Fe_3_O_4_ nanoparticles had mean crystallite diameter of 40.74 nm, which enhances the composite’s surface area and adsorption efficiency. Additionally, a ZnO peak observed at 2θ = 30.46°, corresponding to the (001) plane, confirms its role in pore formation and stabilization^17^. Following Cr(VI) adsorption, the XRD peaks appeared at 2θ = 27.22°, 35.78°, and 62.92°, signifying structural changes in the MBC. These alterations result from chromium species adsorbing onto the composite’s surface, causing minor lattice distortions or strain in the Fe_3_O_4_ crystal structure.

FTIR spectra before and following Cr(VI) removal are illustrated in Fig. 2b. The images exhibit several characteristic peaks corresponding to the functional groups of MBC. The 3737 cm⁻^1^ signal is due to the vibrations of − OH groups, indicating the existence of surface hydroxyl groups^18^. The 3023 cm⁻^1^ signal belongs to the vibrations of -CH groups, suggesting the presence of aromatic or aliphatic hydrocarbons within the carbon matrix. A prominent 2355 cm⁻^1^ peak denotes the C ≡ C vibrations, verifying the existence of alkyne groups^19^. 1575 cm⁻^1^ belongs to C = O and C = C vibration, signifying the presence of carbonyl and aromatic groups. 1268 cm⁻^1^ peak is because of C–O vibration, signifying the existence of phenolic or ether groups. Additionally, the 650 cm⁻^1^ signal is due to Fe–O bond, verifying the embedding of Fe_3_O_4_ nanoparticles in the biochar matrix^20^.

Following Cr(VI) adsorption, a new peak was detected at 459 cm⁻^1^, associated with Cr–O bond, validating the attachment of chromium species onto MBC^21^. This peak confirms chromium complex formation, indicating effective Cr(VI) interaction with MBC’s functional groups. The shifts and changes in peak intensities observed after Cr(VI) adsorption suggest that functional moieties namely C = O, -OH, and C–O took part in the Cr(VI) uptake. These groups likely aided Cr(VI) reduction and chromium complex formation.

The thermal stability of the MBC was assessed using TGA, with the weight loss profile presented in Fig. 2c. The TGA curve exhibited three distinct stages of weight loss. The first stage, accounting for approximately 17% weight loss below 101 °C, corresponds to the evaporation of physically adsorbed moisture and volatile compounds^22^. The second major weight loss (~ 15%) occurred between 101 °C and 480 °C, which can be attributed to the thermal degradation of hemicellulose and cellulose components within the MBC matrix^23^. A gradual and continuous weight loss (~ 15%) was observed between 480 °C and 800 °C, associated with the breakdown of more stable carbonaceous structures. By the end of the heating cycle at 800 °C, the MBC retained approximately 53% of its original mass, indicating a significant fraction of thermally stable carbonaceous material.

**Fig. 3:**
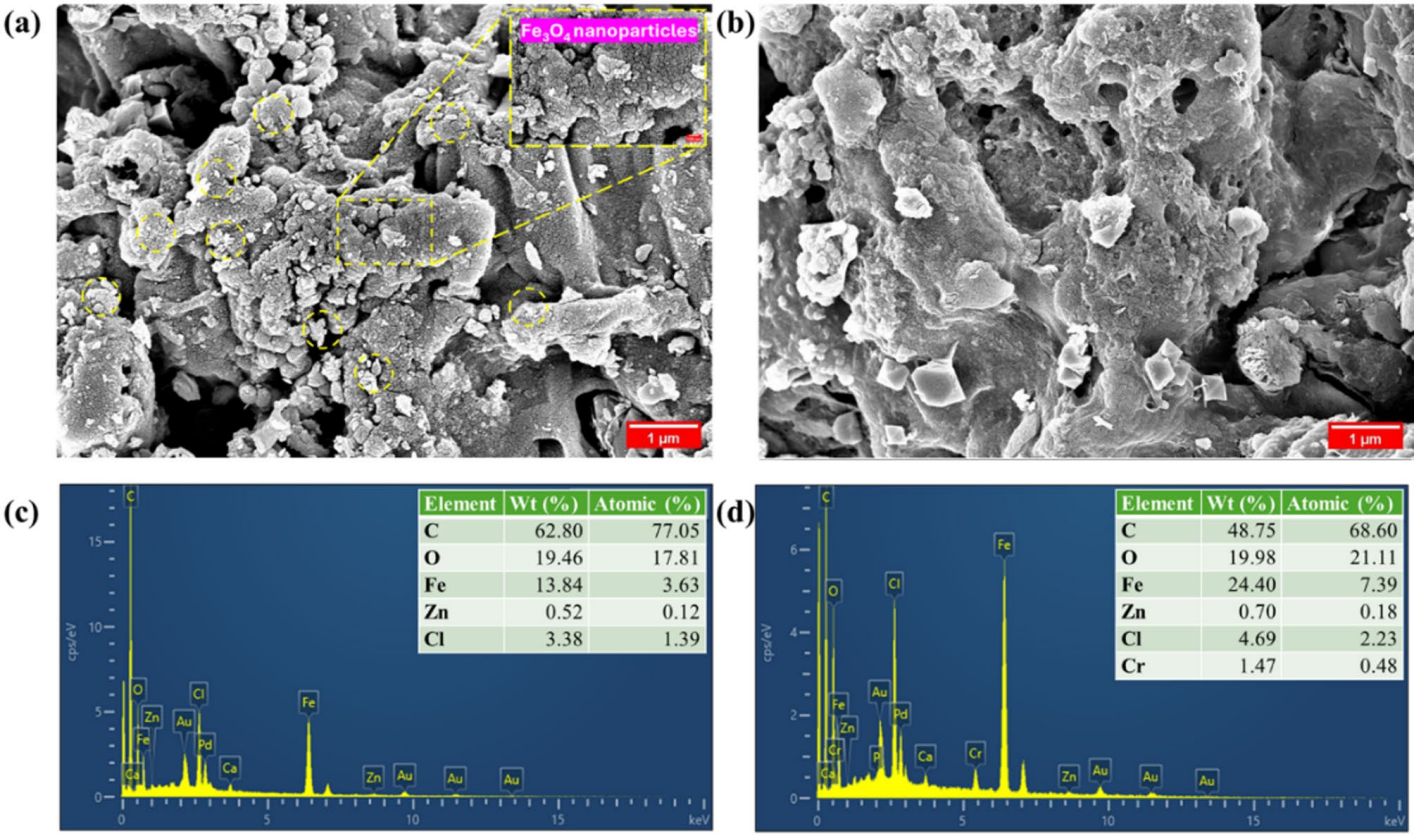
FESEM micrograph (**a**,**b**) and EDS spectra (**c**,**d**) of MBC before and after Cr(VI) adsorption.

The FESEM micrograph before Cr(VI) adsorption (Fig. 3a) reveals a highly porous and rough surface, characteristic of MBC. The porous network enables the swift transport of Cr(VI) to adsorption sites, enhancing the adsorption. The interconnected pores and channels visible in the FESEM image indicate a well-developed hierarchical pore structure, which is advantageous for accommodating adsorbate molecules of different sizes^24^. A closer examination of the magnified region reveals uniformly embedded Fe_3_O_4_ nanoparticles, confirming the composite’s successful formation. ImageJ software was used to determine the average Fe_3_O_4_ nanoparticle size, which was 38.44 nm, confirming the nanoscale. The FESEM image post-Cr(VI) adsorption (Fig. 3b) reveals a denser, smoother surface with reduced pore visibility, indicating partial blockage by Cr(VI) ions. The interconnected pores appear less prominent, suggesting decreased porosity due to Cr(VI) accumulation. These changes confirm effective Cr(VI) adsorption onto MBC^25^.

The pre-adsorption EDS spectra (Fig. 3c) confirm the presence of oxygen, carbon, iron, zinc, and chlorine in the MBC composite. The high carbon content (77.05%) and the presence of Fe (3.63%) and Zn (0.12%) indicate the successful doping of Fe and Zn into the carbon matrix. Post-Cr(VI) adsorption (Fig. 3d), notable alterations in elemental composition are evident. Two distinct peaks corresponding to chromium at 0.57 and 5.41 keV confirm the adsorption of chromium species onto the MBC^26^. Elemental analysis shows 0.48% chromium, indicating effective chromium uptake by the MBC.

The pore volume of MBC was 0.1616 cm^3^/g, with a mean diameter of 2.61 nm and a high specific surface area (SSA) of 248.11 m^2^/g. In contrast, the SSA of other adsorbents is lower, such as spruce sawdust derived MBC (93 m^2^/g)^27^, and palm oil empty bunch MBC (25.79 m^2^/g)^28^. The high SSA offers abundant binding sites for Cr(VI), alongside, the mesoporous structure aids chromium diffusion into internal pores. Post-Cr(VI) adsorption, SSA dropped to 170.78 m^2^/g, with pore volume and size reducing to 0.0681 cm^3^/g and 1.59 nm, respectively. The substantial decrease in SSA and pore volume indicates that Cr(VI) species were effectively trapped within the pores of the MBC and the BET findings aligned with the FESEM analysis results.

**Fig. 4:**
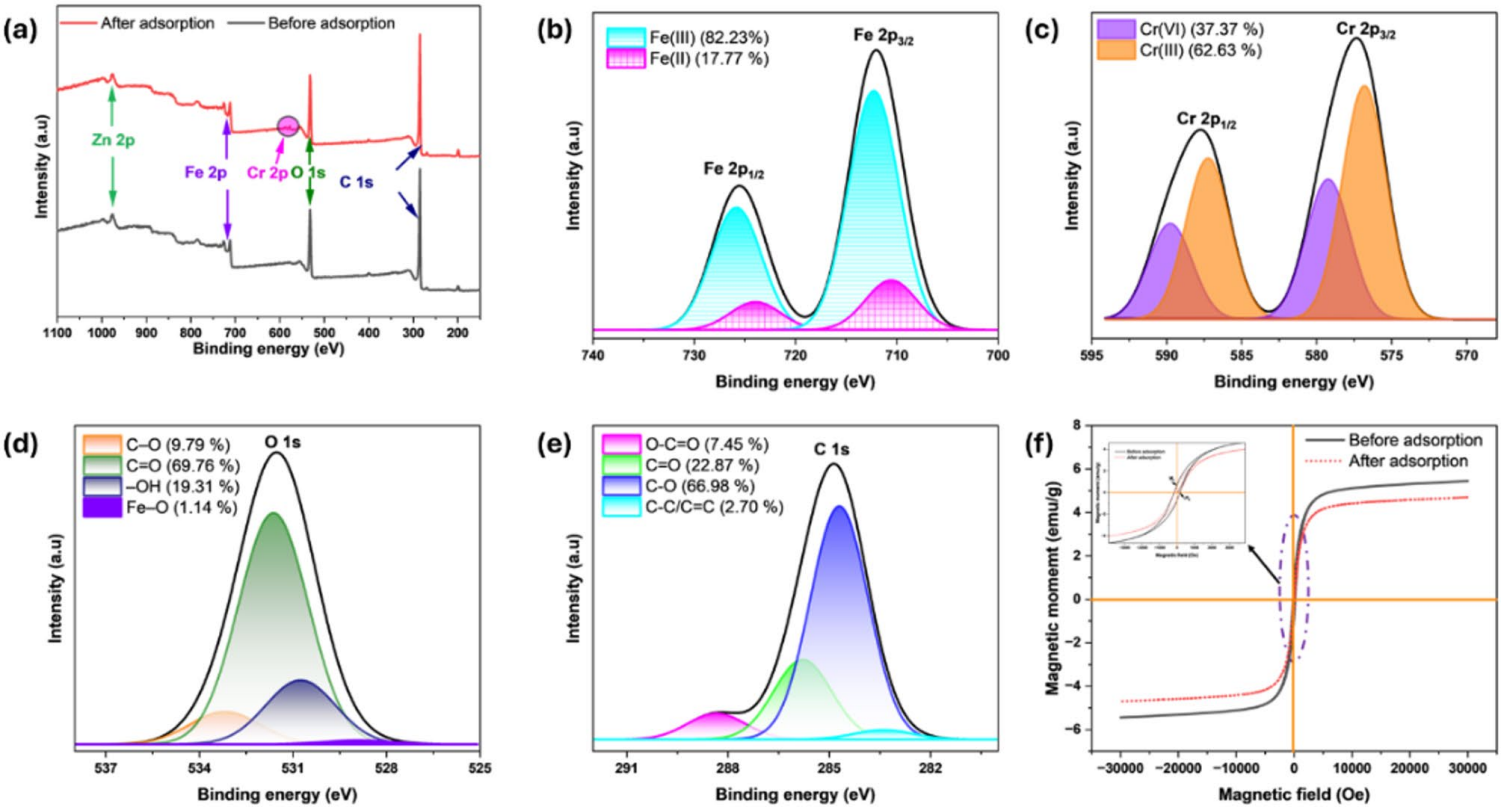
XPS (**a**–**e**) and VSM analysis (**f**) of MBC before and after Cr(VI) adsorption.

Surface analysis via XPS examined the surface composition and oxidation states of MBC before and following Cr(VI) uptake. The complete survey of the adsorbent before Cr(VI) adsorption (Fig. 4a) detected C1s at 285.23 eV, O1s at 531.89 eV, Fe2p_3/2_ at 711.92 eV, and Fe2p_1/2_ at 726.19 eV, verifying the coexistence of carbon, oxygen, and iron on MBC^29^. The Zn 2p peaks at 977.08 eV (Zn2p_3/2_) and 995.04 eV (Zn2p_1/2_) confirm the incorporation of Zn from ZnCl_2_^5^. Zinc promoted biomass dehydration and aromatization, enhancing pore development and surface area. Following Cr(VI) adsorption, the XPS peaks for C1s, O1s, Fe2p_3/2_, and Fe2p_1/2_ showed slight shifts, indicating electronic environment changes due to interactions with Cr(VI). Additionally, two new peaks corresponding to Cr2p_3/2_ at 577.22 and Cr2p_1/2_ at 587.68 eV were observed, validating the successful uptake of chromium species onto the MBC surface.

Figure 4b–e illustrates the deconvoluted XPS spectrum following Cr(VI) removal on MBC. The Fe2p spectrum (Fig. 4b) verifies the existence of both Fe(III) and Fe(II) oxidation states. Peaks at 725.84 and 712.28 eV relate to Fe(III), while those at 723.95 and 710.56 eV belong to Fe(II). The distribution shows that Fe(III) is the dominant oxidation state, comprising 82.23%, whereas Fe(II) accounts for 17.77%. The presence of Fe(II) is crucial for Cr(VI) reduction, functioning as electron donor for facilitating its conversion to Cr(III)^30^. Furthermore, this confirms the presence of Fe_3_O_4_ nanoparticles and corroborates the XRD results. The Cr2p spectrum (Fig. 4c) shows two pairs of peaks corresponding to Cr(VI) and Cr(III) oxidation states. The existence of adsorbed Cr(VI) species is indicated by peaks at 589.75 eV and 579.23 eV, while peaks at 587.23 eV and 576.81 eV confirm the Cr(VI) conversion to Cr(III) on MBC. Cr(VI) constitutes 37.37% of the total chromium, whereas Cr(III) accounts for 62.63%, demonstrating a substantial conversion of Cr(VI)^31^. This reduction is enabled by surface functional moieties on the MBC, supporting a redox-coupled Cr(VI) removal mechanism and complexation with surface functional moieties^32^. The O1s spectrum (Fig. 4d) shows peaks at 531.62 eV for C = O, 530.75 eV for –OH, 533.18 eV for C–O, and 528.91 eV for Fe–O, which facilitate Cr(VI) uptake through electrostatic attraction and redox interactions. The C1s spectrum (Fig. 4e) displays peaks at 284.70 eV for C–O, 285.78 eV for C = O, 288.34 eV for O–C = O, and 283.39 eV for C–C/C = C, suggesting the involvement of hydroxyl, carbonyl, and aromatic structures in Cr(VI) removal via hydrogen bonding and redox reactions^33^.

MBC’s magnetic properties were assessed using VSM analysis, before and post Cr(VI) uptake, as shown in Fig. 4f. The magnetization curves exhibit an S-shaped curve with superparamagnetic behavior and no significant hysteresis loop, indicating the absence of permanent magnetism^34^. Magnetic saturation (M_s_) decreased from 5.44 to 4.67 emu/g after Cr(VI) adsorption due to surface coverage of magnetic active sites by Cr(VI) ions, which interacted with Fe_3_O_4_ nanoparticles, leading to Cr(VI) reduction and surface complexation^35^. The inset of Fig. 4f shows slight changes in remanence magnetization (M_r_) from 0.82 to 0.62 emu/g and coercivity (H_c_) from 35.08 to 41.53 O_e_, suggesting Cr(VI) ion interactions with magnetite nanoparticles. The minimal M_r_ and H_c_ values, along with the absence of a significant hysteresis loop, confirm the superparamagnetic nature of the material. This superparamagnetic behavior prevents agglomeration, ensures easy dispersion, retains a high surface area for Cr(VI) adsorption, and enables efficient magnetic recovery, making it suitable for Cr(VI) remediation^36^. Furthermore, the M_s_ of MBC (5.44 emu/g) synthesized in this study is higher than that of other adsorbents, such as macadamia MBC (2.56 emu/g)^37^, coconut haustorium MBC (2.4 emu/g)^38^, and *Vateria indica* MBC (4.74 emu/g)^39^.

### Adsorption evaluation

#### Role of operating conditions

**Fig. 5:**
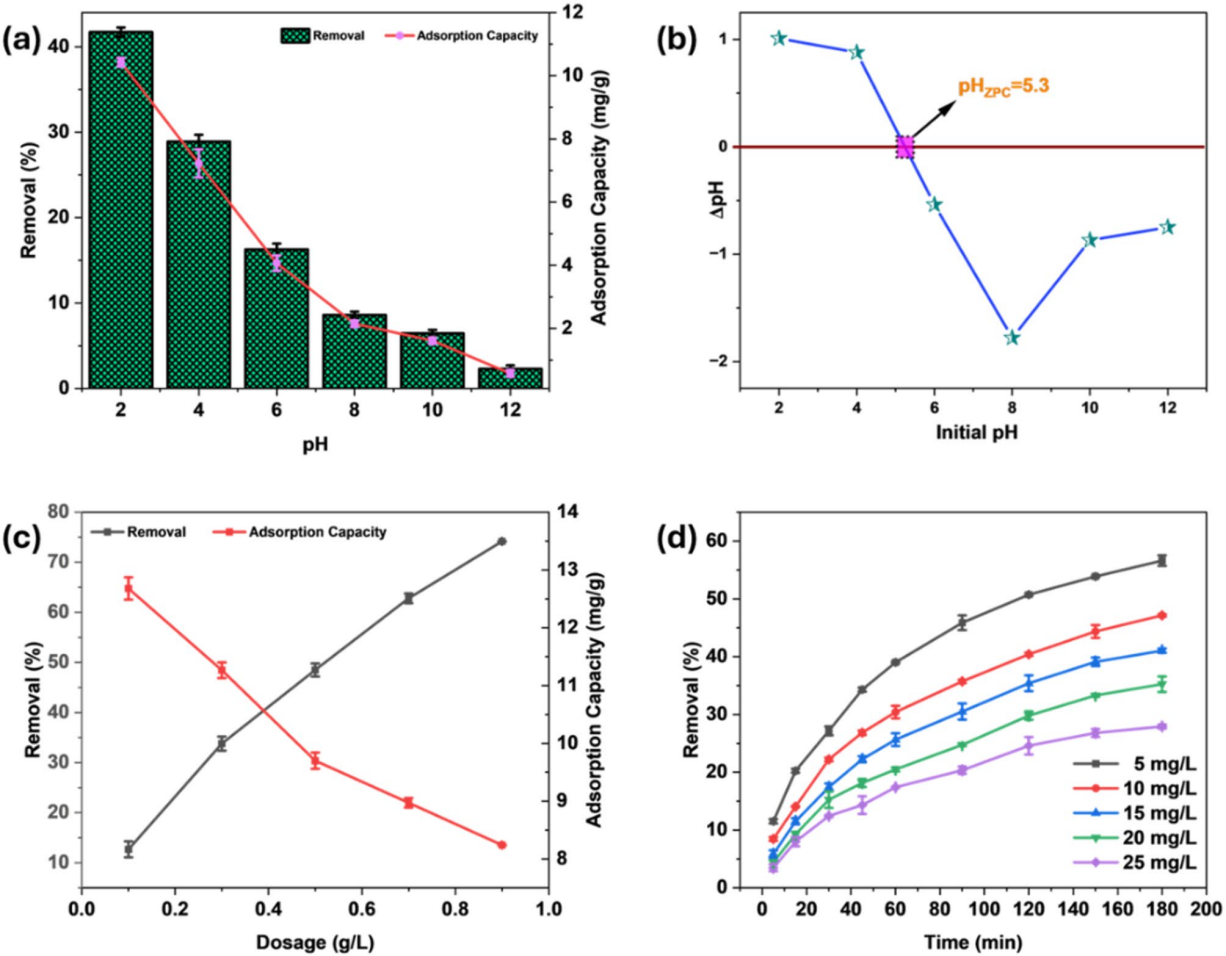
Effect of pH (dose: 0.4 g/L, C_0_: 10 mg/L, T: 303 K, t: 3 h, V: 100 ml) (**a**), zero point charge of MBC (**b**), MBC dose (pH: 2, C_0_: 10 mg/L, T: 303 K, t: 3 h, V: 100 ml) (**c**), Cr(VI) initial concentration and time (dose: 0.4 g/L, pH: 2, T: 303 K, V: 100 ml) (**d**).

The solution pH substantially influenced the Cr(VI) removal potential of the MBC. With the increase in pH from 2 to 12, both adsorption capacity and removal efficiency showed a declining trend (Fig. 5a). At pH 2, the MBC achieved the maximum Cr(VI) uptake of 41.72%, with a corresponding adsorption potential of 10.43 mg/g. However, with increasing pH, the removal performance decreased, hitting a low of 2.31% at pH 12, while adsorption capacity declined to 0.57 mg/g. The observed trend results from the pH-dependent Cr(VI) speciation, MBC’s surface charge characteristics, and the redox potential of Cr(VI) species. In acidic media, Cr(VI) predominantly appears as HCrO_4_⁻ and Cr_2_O_7_^2^⁻, forming strong electrostatic interactions with protonated sites namely –OH and –COOH on the MBC surface, enhancing adsorption. At lower pH, Cr(VI) exhibits a higher redox potential, promoting its reduction to Cr(III), which enhances removal by precipitating as Cr(OH)_3_ and Cr_2_O_3_ on the MBC^40^.

However, as the pH rises, deprotonation of carboxyl groups and hydroxyl creates more surface negativity, inducing electrostatic repulsion with the CrO_4_^2^⁻. Furthermore, excess hydroxide ions (OH⁻) compete against Cr(VI) anions for available adsorption sites, further reducing removal efficiency. Additionally, at higher pH values, Cr(VI) exhibits a lower redox potential, diminishing its tendency to reduce to Cr(III). Furthermore, Cr(III) may undergo hydrolysis and subsequently precipitate as Cr(OH)_3_ and Cr_2_O_3_^29^. Comparable findings were observed for Cr(VI) adsorption using adsorbents like *Moringa oleifera* AC^41^, and bone waste magnetic AC^42^.

As depicted in Fig. 5b, MBC’s zero point charge (pH_ZPC_) was identified as 5.3. At pH levels below 5.3, the positive surface charge promotes electrostatic attraction with Cr(VI) species (HCrO_4_⁻, Cr_2_O_7_^2^⁻) and facilitates its reduction to Cr(III), enhancing removal efficiency^43^. Above pH 5.3, surface deprotonation led to electrostatic repulsion with CrO_4_^2^⁻, reduced Cr(VI) redox potential, and competition from OH⁻ ions, lowering adsorption. Thus, Cr(VI) removal was most effective under acidic conditions. These findings are supported by pH-dependent adsorption studies, which revealed a notable decrease in adsorption efficiency with rising solution pH^44,45^.

Figure 5c depicts the dosage effect from 0.1 to 0.9 g/L at a pH of 2 and concentration of 10 mg/L. As the dosage raised from 0.1 to 0.9 g/L, the adsorption efficiency improved from 12.68 to 74.15%, because of the large SSA and binding spots, which facilitated enhanced Cr(VI) removal. However, adsorption capacity declined from 12.67 to 8.23 mg/g due to a large number of unsaturated binding sites at higher doses. At lower dosages, the limited availability of binding sites and less surface area resulted in higher adsorption capacity, whereas at higher dosages, excess MBC led to the presence of unoccupied binding sites, reducing the adsorption capacity^46^. This inverse relationship aligns with previously reported adsorption studies^47^, emphasizing that while a higher MBC dose enhances removal efficiency, it diminishes adsorption capacity due to the saturation of available chromium ions and underutilization of active sites. Optimizing the MBC dose enhances cost-effective wastewater treatment by maximizing adsorption. Thus, 0.4 g/L MBC concentration was employed for further batch adsorption studies.

MBC was tested for Cr(VI) removal under optimum conditions by changing the Cr(VI) concentration from 5 to 25 mg/L, as depicted in Fig. 5d. The maximum Cr(VI) adsorption of 56.62% was observed at 5 mg/L, whereas the lowest removal of 27.91% was observed at 25 mg/L. Elevated Cr(VI) concentrations lead to a decline in adsorption efficiency due to the saturation of active spots on the MBC. Although elevated Cr(VI) levels enhance bulk diffusion and increase the driving force for adsorption, the limited number of active sites restricts further adsorption, leading to a lower overall removal performance^48^. Whereas, at lower concentrations, ample binding sites on the MBC surface remain available, allowing efficient Cr(VI) uptake and enhancing removal efficiency. These findings highlight the significance of optimizing the initial Cr(VI) level to enhance adsorption performance and ensure maximum adsorbent utilization. For subsequent experiments, Cr(VI) level of 10 mg/L was chosen.

Figure 5d demonstrates a characteristic trend typically observed in batch adsorption processes for the dependence of time. Initially, quick removal of Cr(VI) is observed during the initial 30 min for all initial concentrations of Cr(VI). This swift removal occurs because of the steep concentration difference between the bulk solution and the MBC surface, promoting the rapid diffusion of Cr(VI) to the binding sites on MBC^45^. At this early phase, numerous unoccupied binding sites on MBC facilitate effective adsorption^48^. As adsorption time increases, the Cr(VI) removal rate slows after 30 min and stabilizes by 180 min. This occurs because of the decline in Cr(VI) species in the solution, which reduces the concentration gradient and adsorption driving force, along with the gradual saturation of binding sites on the MBC. By 180 min, the adsorption process reaches a plateau, indicating that the adsorption sites are nearing saturation, and the system is approaching equilibrium. These findings indicate that 180 min represents the ideal duration for achieving maximum Cr(VI) removal, ensuring efficient Cr(VI) remediation.

#### Study of adsorption models

**Fig. 6:**
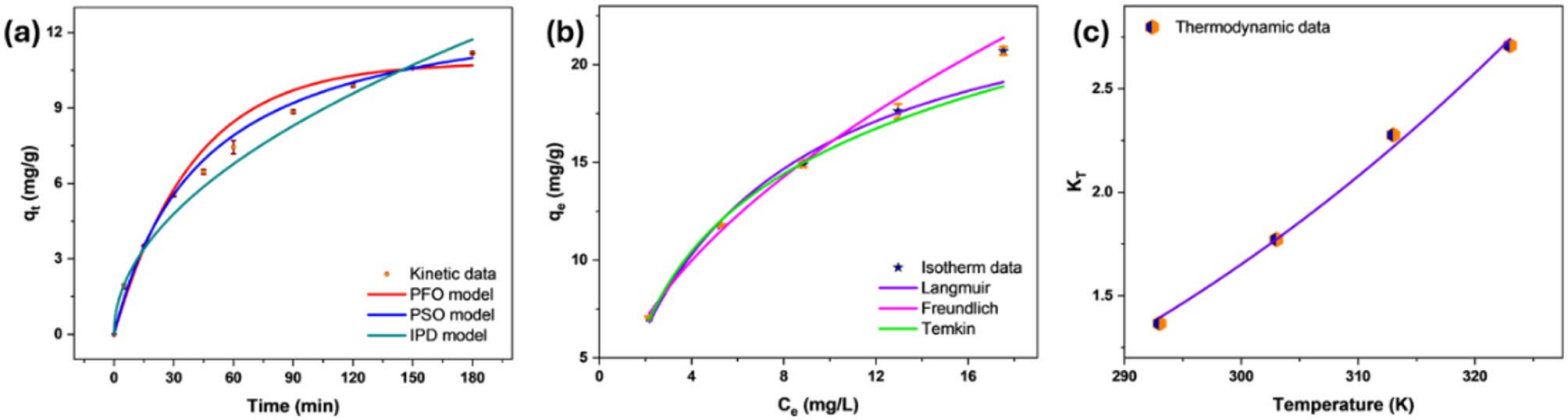
Kinetic models (dose: 0.4 g/L, pH: 2, C_0_: 10 mg/L, T: 303 K, t: 3 h, V: 100 ml) (**a**), isotherm models (dose: 0.4 g/L, pH: 2, T: 303 K, t: 3 h, V: 100 ml) (**b**) and thermodynamic model (dose: 0.4 g/L, pH: 2, C_0_: 10 mg/L, t: 3 h, V: 100 ml) (**c**) of Cr(VI) removal using MBC.

The Cr(VI) adsorption process was analyzed employing kinetic models namely PFO, PSO, and IPD, with results illustrated in Fig. 6a and model parameters summarized in Table 1. The kinetic models demonstrated that the PSO model was the most suitable, with R^2^ = 0.994 and χ^2^ = 0.083, followed by the IPD (R^2^ = 0.988, χ^2^ = 0.193), and the PSO model (R^2^ = 0.985, χ^2^ = 0.229). The better correlation with the PSO model denotes that chemisorption dominates the Cr(VI) removal, relying on valence forces through electron sharing between Cr(VI) and MBC’s functional groups. The adsorption capacity showed an initial rapid increase within the first 30 min due to bulk diffusion, intraparticle transport, and pore-filling effects^49^. This phase was enhanced by the abundance of reactive sites and extensive surface availability, promoting Cr(VI) binding. As adsorption progressed, the rate declined, indicating a transition from surface adsorption to Cr(VI) diffusion into the micropores, leading to equilibrium^50^. Intra-particle diffusion influences the adsorption but does not solely determine the rate-controlling step, as suggested by the IPD model. Instead, adsorption occurs through a combination of surface interaction and pore diffusion. The data validate earlier findings, indicating that the chemisorption mechanism significantly influences the Cr(VI) removal^25,51^.

**Table 1:** Kinetic, isotherm and thermodynamic model parameters of Cr(VI) adsorption of MBC.

Kinetic models		
Kinetic model	Equation	Parameters
Pseudo first order	$\:{q}_{t}=\:{q}_{e\:}\left(1-exp\left({-k}_{1}t\right)\right)$	$\:{k}_{1}$ (min^− 1^) *=* 0.02
		$\:{q}_{e}$ (mg/g) *=* 10.95
		*R*^*2*^ *=* 0.985 χ^2^ = 0.229
Pseudo second order	$\:{q}_{t}=\frac{{q}_{e}^{2}{k}_{2}t}{{{q}_{e}K}_{2}t+1}$	$\:{k}_{2}$ (g/mg.min) *=* 0.0015
		$\:{q}_{e}$, (mg/g) = 13.82
		*R*^*2*^ = 0.994 χ^2^ = 0.083
Intra-particle diffusion	$\:{q}_{t}={k}_{p}{t}^{0.5}$+$\:C$	$\:{k}_{p}$ ((mg/g) min^0.5^) = 0.857
		$\:C$ (mg/g) = 0.353
		*R*^*2*^ = 0.988 χ^2^ = 0.193

Isotherm models		
Isotherm	Equation	Parameters
Langmuir	$\:{q}_{e}=\frac{{q}_{max}b{C}_{e}}{{(1+bC}_{e})}$	$\:{q}_{max}$(mg/g) *=* 25.62
		*b* (L/mg) *=* 0.167
		*R*^*2*^ *=* 0.987 χ^2^ = 37.61
Freundlich	$\:{q}_{e}={K}_{F}\:{C}_{e}^{1/n}$	*K*_*F*_ ((mg/g)/(mg/L)^1/n^) = 4.87
		$\:1/n$ = 0.52
		*R*^*2*^ *=* 0.993 χ^2^ = 18.61
Temkin	$\:{q}_{e}=B\:ln\left(A{C}_{e}\right)$	B = 5.71 A (L/mg) = 1.55 R^2^ = 0.988 χ^2^ = 34.92

Thermodynamic model				
T (K)	293	303	313	323
ΔG° (kJ/mol)	− 0.76	− 1.44	− 2.14	− 2.67
	**Equation**	**Parameters**		
	$\:{K}_{T}=\text{exp}\left[\left(\frac{\varDelta\:S^\circ\:}{R}\right)-\left(\frac{\varDelta\:H^\circ\:}{R}\right)\frac{1}{T}\right]$	ΔH° (kJ/mol) = 17.71 ΔS° (J/mol K) = 63.22 *R*^*2*^ *=* 0.995 χ^2^ = 0.002		

q_e_: Equilibrium adsorption capacity; K_1_: PFO constant; K_2_: PSO constant; K: IPD rate constant; C: IPD intercept; q_max_: Monolayer adsorption capacity; b: Langmuir constant; K_F_: Freundlich constant; 1/n: adsorption intensity; C_e_: equilibrium Cr(VI) concentration; A & B: Temkin constants; ΔG°: (= – RT ln K_T_), standard Gibbs free energy; K_T_: (q_e_/C_e_), distribution factor; ΔH°: standard enthalpy and ΔS°: standard entropy.

Freundlich, Langmuir, and Temkin models were executed to fit the experimental data for Cr(VI), as given in Fig. 6b, with model parameters outlined in Table 1. The isotherm trend indicates that the adsorption capacity increases with rising equilibrium concentration. This behavior results from the abundant number of Cr(VI) ions, which strengthens the mass transfer of Cr(VI) to the MBC surface due to the concentration gradient. As the concentration increases, more ions interact with available binding sites, leading to a higher adsorption capacity^52^. Among the models, the Freundlich isotherm exhibited the good fit (R^2^ = 0.993, χ^2^ = 18.61), followed by the Temkin (R^2^ = 0.988, χ^2^ = 34.92) and Langmuir (R^2^ = 0.987, χ^2^ = 37.61) models. The superior fit of the Freundlich model indicates that Cr(VI) binding takes place on a heterogeneous surface with multilayer adsorption, indicating the presence of diverse binding sites with varying affinities^53^. This behavior aligns with the physicochemical properties of the MBC, which offers multiple functional groups that interact with Cr(VI) ions at different energy levels. Furthermore, the maximum adsorption capacity of MBC was found to be 25.62 mg/g, demonstrating superior performance compared to various other adsorbents previously reported in the literature. This enhanced capacity highlights the effectiveness of MBC in Cr(VI) removal and is further supported by the comparative data presented in Table 2.

**Table 2:** Comparative study of Cr(VI) removal efficiency using various adsorbents.

Adsorbent	Synthesis Method	S_a_ (m^2^/g)	pH	T(K)	Dose (g/L)	Time (min)	Cr (VI) (mg/L)	q_max_ (mg/g)	Ref.
Zeolite-based magnetic composite	Co-precipitation	223.85	2	298	1.6	90	–	2.85	^60^
*Schleichera oleosa* magnetic biochar	Co-precipitation	–	2	298	4	80	5 to 25	3.197	^61^
Rice husk magnetic biochar	Pyrolysis	134.6	2	351	0.5	120	1 to 10	9.97	^58^
*E. prolifera* magnetic biochar	Pyrolysis	–	2	298	–	2880	5 to 200	11.13	^62^
*A. falcata* leaves magnetic nanoparticle	One pot synthesis	130.23	2	303	1.5	120	10 to 30	12.91	^44^
Paper waste sludge hydrochar modified Fe(II)	Hydrothermal carbonization	4.16	3	298	1	240	10 to 80	14.33	^63^
Algae magnetic AC	Pyrolysis	–	3	–	1	90	20 to 60	15.24	^64^
Zn-Fe doped Magnetic Biochar	Pyrolysis	248.11	2	303	0.4	180	5 to 25	25.62	This study

Temperature variations from 293 to 323 K were examined in thermodynamic study to assess the feasibility of Cr(VI) uptake. The equilibrium constant (K_T_) plotted against temperature showed a strong fit (R² = 0.995, χ² = 0.002). The trend is depicted in Fig. 6c, while thermodynamic parameters are provided in Table 1. The Gibbs free energy (ΔG°) measurements, ranging from – 0.76 kJ/mol at 293 K to – 2.67 kJ/mol at 323 K, indicated enhanced spontaneity with increasing temperature. This enhanced spontaneity is because of the greater diffusion of Cr(VI) molecules from the bulk solution to the MBC binding sites, facilitated by increased thermal energy^54^. The positive enthalpy (ΔH° = 17.71 kJ/mol) confirmed that the adsorption of Cr(VI) is endothermic, favoring adsorption at elevated temperature^55^. Moreover, the magnitude of ΔH° (≤ 60 kJ/mol) suggested that electrostatic interactions and pore diffusion significantly influence adsorption^56^. The positive entropy (ΔS° = 63.22 J/mol K) indicated enhanced randomness along with increased mobility of Cr(VI) ions^57^.

#### Possible adsorption mechanism

**Fig. 7:**
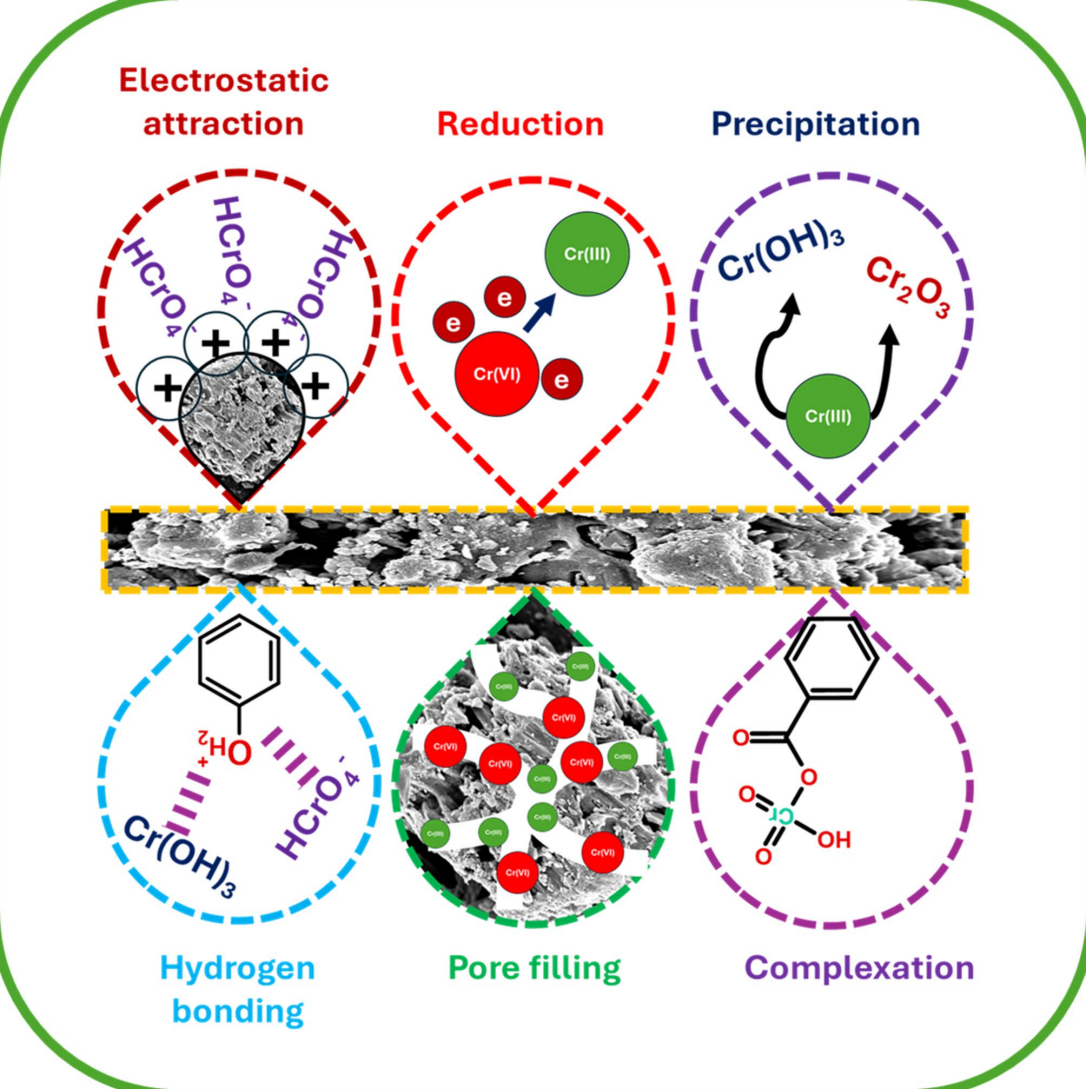
Schematic representation of Cr(VI) removal mechanisms using MBC.

The adsorption of Cr(VI) using MBC includes multiple mechanisms, as presented in Fig. 7. After Cr(VI) uptake, the BET analysis showed a decrease in MBC’s SSA (248.11 to 170.78 m^2^/g), pore volume (0.1616 to 0.0681 cm^3^/g), and pore diameter (2.61 to 1.59 nm), indicating pore blockage by Cr(VI) ions^25^. FESEM analysis further confirmed this by showing a more compact and aggregated morphology due to the deposition of chromium species on MBC. Additionally, EDS analysis detected two distinct peaks at 0.57 and 5.41 keV, confirming the presence of chromium after adsorption^26^.

Electrostatic attraction played a key role, particularly at acidic pH, where protonation of functional groups (-OH and -COOH) facilitated interactions with negatively charged Cr(VI) species (HCrO_4_⁻, Cr_2_O_7_^2^⁻)^58^. Additionally, the higher redox potential of Cr(VI) at acidic environment promoted its conversion to Cr(III), subsequently precipitating as Cr(OH)_3_ and Cr_2_O_3_ on the MBC, thereby enhancing removal^40^. XRD analysis revealed peak shifts after Cr(VI) adsorption, suggesting structural modifications in the Fe_3_O_4_ crystal lattice, likely caused by interactions between chromium species and Fe_3_O_4_ nanoparticles. FTIR analysis identified a new peak at 459 cm⁻^1^, due to Cr–O vibrations, confirming the formation of chromium complexes on MBC^21^. Shifts and intensity variations in functional groups namely as -OH, C = O, and C–O further suggested their involvement in the Cr(VI) adsorption, facilitating the reduction.

XPS analysis provided further insights into the redox mechanism. The Fe2p spectrum revealed both Fe(III) and Fe(II), emphasizing the involvement of Fe(II) as an electron donor in the Cr(VI) reduction to Cr(III)^30^. The C1s and O1s spectra of MBC display peaks pertaining to C–C/C = C, C–O, C = O, and O–C = O, confirming the existence of aromatic, hydroxyl, carbonyl, and carboxyl functional moieties^33^. The Cr2p spectrum exhibited peaks corresponding to both Cr(VI) (37.37%) and Cr(III) (62.63%), confirming the substantial conversion of Cr(VI) during adsorption^32^. This reduction, coupled with complexation, facilitated Cr(VI) adsorption onto the MBC surface. VSM analysis showed a decrease in magnetic saturation (M_s_) from 5.44 to 4.67 emu/g after Cr(VI) uptake, confirming that Cr(VI) ions occupied the magnetic sites, prompting surface complexation with Fe_3_O_4_ nanoparticles^35^.

#### Regeneration studies

**Fig. 8:**
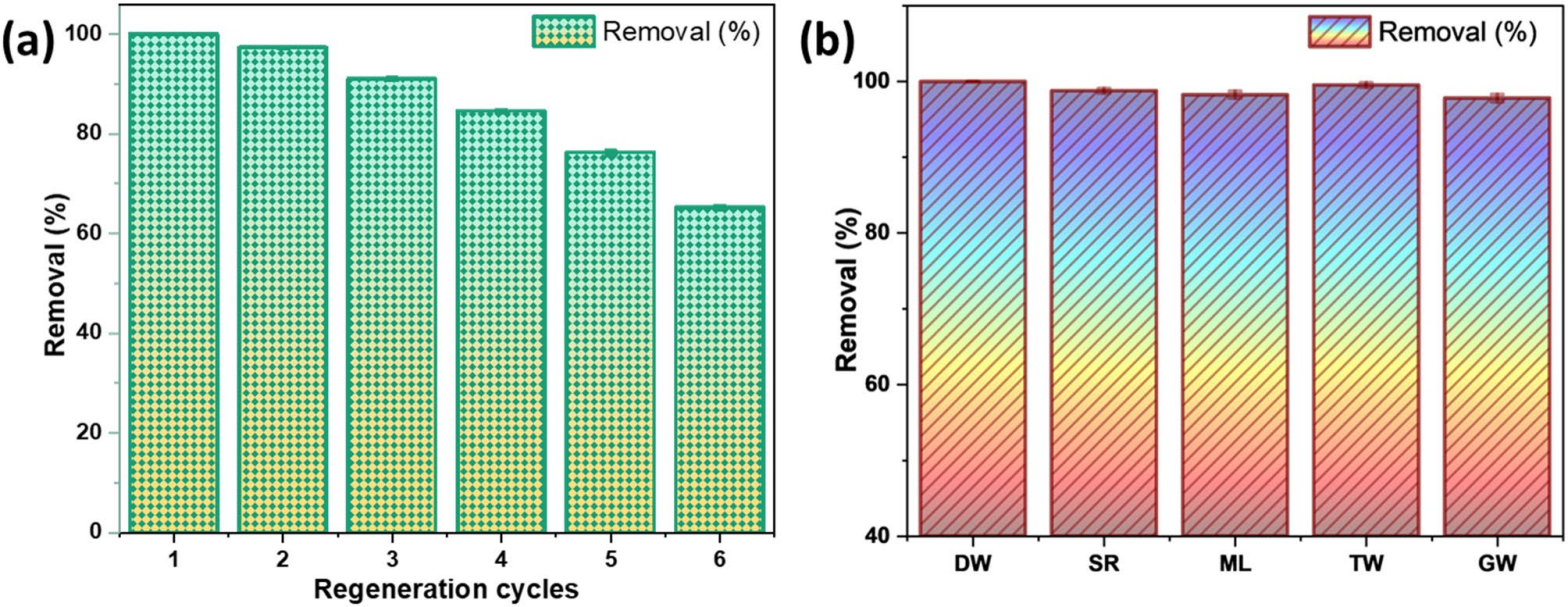
Regeneration (dose: 1.4 g/L, pH: 2, C_0_: 10 mg/L, T: 303 K, t: 3 h, V: 100 ml) (**a**) and spiked Cr(VI) removal efficiency (dose: 1.4 g/L, pH: 2, C_0_: 10 mg/L, T: 303 K, t: 3 h, V: 100 ml) (**b**) using MBC.

The regeneration process is important for the sustainability and cost-effectiveness of Cr(VI) remediation, as it allows adsorbents to be reused multiple times, reducing material consumption and treatment costs. The results, as given in Fig. 8a, indicate a progressive decline in Cr(VI) removal with each regeneration cycle. The adsorption-desorption study using 0.1 N NaOH showed a decrease in efficiency from 99.98% in the first cycle to 76.21% in the fifth cycle, maintaining effective performance. However, by the sixth cycle, it dropped to 65.20%, indicating a significant reduction in adsorption capacity due to pore blockage, structural changes, and the loss of active functional groups^59^. Since the removal efficiency remained above 76% up to the fifth cycle, MBC can be effectively reused a maximum of five times before experiencing a substantial decline. Beyond this, its regeneration potential weakens, limiting long-term applicability. Overall, MBC demonstrates good reusability, making it a viable and economical option for Cr(VI) removal. However, further optimization strategies, such as modifying regeneration conditions or enhancing adsorbent stability, may be required to improve long-term performance and extend its usability in wastewater treatment.

#### Evaluation of Cr(VI) remediation in natural water matrices

Spiked adsorption studies were done to explore the effect of interfering ions in different water matrices on Cr(VI) adsorption efficiency. Swarna River, Manipal Lake, tap water, and groundwater were used to make a 10 mg/L Cr(VI) aqueous media for batch experiments with a 100 mL sample volume. The results were compared with distilled water as a reference. The Cr(VI) removal percentages for different water sources are illustrated in Fig. 8b. The findings indicate that Cr(VI) removal was highest in distilled water (99.99%), followed by tap water (99.53%), Swarna River (98.77%), Manipal Lake (98.25%), and groundwater (97.80%). The superior removal in distilled water is due to the lack of interfering ions, whereas the marginally reduced Cr(VI) adsorption in groundwater results from the presence of multiple competing ions. Overall, MBC exhibited strong potential for Cr(VI) removal, achieving an efficiency of over 97.80% across different water matrices.

## Conclusions

MBC was successfully synthesized in a tubular furnace and extensively characterized before and after Cr(VI) removal. FESEM revealed a smoother, more compact surface after Cr(VI) adsorption, consistent with BET analysis, which showed a reduction in surface area from 248.11 to 170.78 m²/g due to pore filling. EDS confirmed the presence of chromium peaks at 0.57 and 5.41 keV, indicating chromium uptake. XRD confirmed graphitic carbon and Fe_3_O_4_ phases, with a cubic spinel structure, a lattice parameter of 0.841 nm, and a crystallite size of 40.74 nm. XPS analysis validated Fe(II) and surface functional groups’ role in reducing Cr(VI) to Cr(III). VSM confirmed Fe_3_O_4_’s superparamagnetic behavior. TGA demonstrated the thermal stability of MBC and indicated a high proportion of thermally stable carbonaceous content.

Batch studies determined an optimum pH of 2, an MBC dose of 0.4 g/L, and a contact time of 3 h, with a maximum adsorption capacity of 25.62 mg/g. The PSO model best described the adsorption process, suggesting electron transfer and sharing plays an important role in Cr(VI) removal. Freundlich isotherm analysis indicated heterogeneous adsorption. The thermodynamic study confirmed the spontaneity and endothermic adsorption of Cr(VI). Regeneration studies showed MBC maintained effective Cr(VI) removal for up to five cycles. The adsorbent achieved over 97.80% Cr(VI) adsorption from groundwater, lake water, and river water, demonstrating its practical potential.

## Data Availability

The data supporting the findings of this study are included within the article.

## Declarations

### Permissions to collect the plants/plant parts

As the tree is abundant and not under protection, no permits or licenses were required for its collection. Additionally, a voucher specimen was not stored in the herbarium due to its broad recognition and availability.

### Plant guidelines

The authors affirm that the use of plant materials in this study follows all applicable international, national, and institutional regulations.

### Source of the plant used in your study

All plant names and their sources are provided in Methodology.
